# Toxicity of persistent organic pollutants: a theoretical study

**DOI:** 10.1007/s00894-024-05890-8

**Published:** 2024-03-07

**Authors:** Ana Martínez

**Affiliations:** Departamento de Materiales de Baja Dimensionalidad, Instituto de Investigaciones en Materiales, Universidad Nacional Autónoma de México, Circuito Exterior S.N. Ciudad Universitaria, 04510 CDMX, CP Mexico

**Keywords:** DAM, PCB-77, PBDE-47, Electron transfer, Carcinogenic, Penguins

## Abstract

**Context:**

Polychlorinated biphenyls (PCBs) and polybrominated diphenyl ethers (PBDEs) are two families of persistent organic pollutants that are dangerous as they remain in the atmosphere for long periods and are toxic for humans and animals. They are found all over the world, including the penguins of Antarctica. One of the mechanisms that explains the toxicity of these compounds is related to oxidative stress. The main idea of this theoretical research is to use conceptual density functional theory as a theory of chemical reactivity to analyze the oxidative stress that PCBs and PBDEs can produce. The electron transfer properties as well as the interaction with DNA nitrogenous bases of nine PCBs and ten PBDEs found in Antarctic penguins are investigated. From this study, it can be concluded that compounds with more chlorine or bromine atoms are more oxidizing and produce more oxidative stress. These molecules also interact directly with the nitrogenous bases of DNA, forming hydrogen bonds, and this may be an explanation for the toxicity. Since quinone-type metabolites of PCBs and PBDEs can cause neurotoxicity, examples of quinones are also investigated. Condensed Fukui functions are included to analyze local reactivity. These results are important as the reactivity of these compounds helps to explain the toxicity of PCBs and PBDEs.

**Methods:**

All DFT computations were performed using Gaussian16 at M06-2x/6–311 + g(2d,p) level of theory without symmetry constraints. Electro-donating (ω-) and electro-accepting (ω +) powers were used as global response functions and condensed Fukui functions as local parameters of reactivity.

## Introduction

Persistent organic pollutants are chemical compounds that remain intact in the environment for long periods [1]. They are widely distributed throughout the world, can accumulate in the fatty tissues of animals, and are toxic and dangerous to humans and wildlife. They are listed in the Stockholm Convention [2] which is a multilateral environmental agreement to “protect human health and the environment from these pollutants.” There are 152 countries that sign this agreement, and the idea is to work together in order to achieve a future free of persistent organic pollutants. In the meantime, it is necessary to know what we can expect from these compounds in relation to human and animal health.

Polychlorinated biphenyls (PCBs) and polybrominated diphenyl ethers (PBDEs) are two families of persistent organic pollutants found in two penguin species in Antarctica [3]. PCBs have two fused benzene rings, with different chlorine atoms replacing the hydrogens. The number and position of chlorine atoms result in 209 different congeners [4]. PCBs have been used as flame retardant products and plasticizers [5]. PBDEs have two benzene rings connected by one oxygen atom. They are organo-brominated compounds in which different bromine atoms replace the hydrogens. The number and position of bromine atoms give rise to different congeners. They are widely used since the 1970s in paints, plastics, textiles, rugs, building materials, airplanes, and automobiles since the 1970s [6, 7]. Constitute up to 30% of these products by weight. Both families of compounds reach the environment and remain for a long time. Degradation takes more than 40 years [1]. Human contact comes from ingestion of contaminated food or air. Exposure to PCBs and PBDEs is associated with neurotoxicity, endocrine dysfunction, and reproductive disorders [8–35]. These compounds are chemically and enzymatically oxidized to active quinone-type derivatives that are also very toxic [36–39]. Quinones are Michael acceptors that can react with glutathione (GSH), proteins, and DNA [37, 38] inducing damage. They also have redox properties that increases oxidative stress [39].

**Fig. 1:**
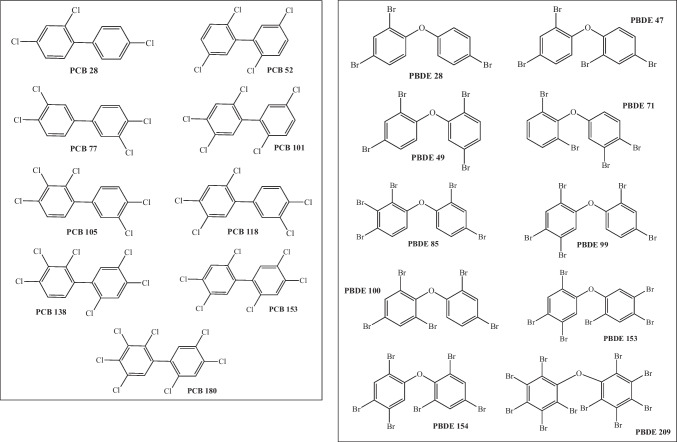
Schematic representation of the studied compounds

Persistent organic pollutants studied in this investigation are those reported in Fig. 1. Within the PCBs, the most toxic are those with *meta*-chlorine and *para*-chlorine substituents [5]. Toxicological studies have shown that non-coplanar PCBs have high reactivity and toxicity [12] with PCB-77 being one of the most toxic [4]. Studies have reported that PCBs increase oxidative stress in the brain-producing apoptosis. This loss of neurons leads to various neurodegenerative diseases [10, 11, 16]. Among PBDEs, the congeners that induce oxidative DNA damage are PBDE-47 and PBDE-209. Within these, PDBE-47 is the most potent [13, 14]. Both compounds were reported to possibly damage DNA through more than one mechanism. Due to its proven toxicity, the production of PCBs and PBDEs has been prohibited for more than 5 years, but the problem is that those that have already been produced remain in the environment.

In Antarctica, *Pygoscelis* penguins are the most abundant vertebrates and are considered good bioindicators of different pollutants [3, 40–43]. In a previous work [3], the levels of PCBs and PBDEs were reported in samples from chinstrap penguin (*Pygoscelis antarcticus*) and gentoo penguin (*Pygoscelis papua*). The most abundant contaminants they found are those presented in Fig. 1. Although penguins are found in Antarctica, an area with a low population and far from civilization, we can find these contaminants. PCBs and PBDEs are not produced or used in this area, but they arrive through air and water. It is important to study these pollutants since they can apparently travel all over the world. Despite all the reported information on the effects on human and animal health produced of PCBs and PBDEs, there are no theoretical studies on the reactivity of these compounds and the oxidative stress that they can produce. There are theoretical investigations concerning the reactivity of PCB-77 [5, 44] that indicate the reaction of these compounds with ·OH during degradation and also regarding the interaction with nucleic DNA nitrogen bases. There are also reports that link oxidative stress to cancer and the development of brain disorders [45–47], but there are not a theoretical investigation concerning the charge transfer process of these compounds. For this reason, the main idea is to analyze the electron transfer process of PCBs and PBDEs. Examples of quinone-type metabolites are also included. Electron donor and acceptor properties of compounds shown in Fig. 1 and quinone-type derivatives of PCB-77 and PBDE-49 are investigated using density functional theory. The interaction of these compounds with DNA nitrogen bases is also analyzed. Quinones-type derivatives of PCB-77 and PBDE-49 were selected since the synthesis of these two quinones was previously reported [36–38]. The criterion for selecting these compounds is that they were found in Antarctic penguins and have been published as toxic. This research provides information on the reactivity of these compounds that may be useful to analyze their potential toxicity in animals and plants.

## Computational details

Gaussian16 was used for all electronic calculations [48]. Geometry optimizations of initial geometries were obtained at M06-2x/6–311 + g(2d,p) level of theory without symmetry constraints [49–51]. These exchange correlation functionals were used before with success. This is a global hybrid functional with 54% HF exchange, and it is the best within the 06 functionals for main group thermochemistry, kinetics, and non-covalent interactions. To search for the global minimum structures, the structures published in PubChem were used as initial geometries. In some examples, we calculated different conformations, and the energy differences between optimized structures were very small. Furthermore, global reactivity descriptors were similar for each conformation. All optimized structures reported here are global minima and do not have negative harmonic frequencies.

Conceptual density functional theory is a chemical reactivity theory founded on density functional theory-based concepts [52–58]. Within this theory, there are global response functions such as the electro-donating (ω-) and electro-accepting (ω +) powers, previously reported by Gázquez and co-workers [53, 54]. The capacity to donate electrons (ω-) or accept electrons (ω +) is defined as follows:

$$\begin{array}{cc}\upomega - ={\left(3\mathrm{I }+ A\right)}^{2} / 16 (I-A)&\upomega + = {(I + 3A)}^{2}/ 16 (I-A)\end{array}$$

*I* and *A* are vertical ionization energy and vertical electron affinity, respectively. They are obtained as follows:

$$\begin{array}{cc}{\mathrm{A}}\to {{\mathrm{A}}}^{+1}+1{\mathrm{e}}& \mathrm{I }=\mathrm{ E }\left({{\mathrm{A}}}^{+1}\right)-\mathrm{E }({\mathrm{A}})\\ {{\mathrm{A}}}^{-1} \to {\mathrm{A}}+1{\mathrm{e}}& \mathrm{A }=\mathrm{ E }\left({\mathrm{A}}\right)-\mathrm{E }({{\mathrm{A}}}^{-1})\end{array}$$

**Fig. 2:**
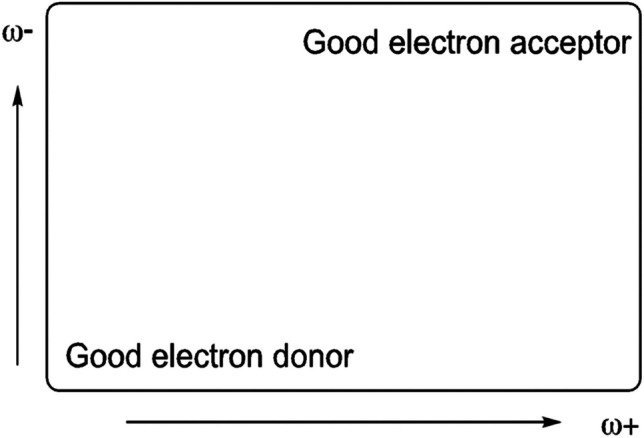
Electron donor–acceptor map (DAM)

Low values of ω- indicate good electron donor molecules. High values of ω + are for good electron acceptor molecules. With these parameters, it is possible to determine the electron donor–acceptor map (DAM, see Fig. 2) [59]. Systems located down to the left are considered good electron donors, while those situated up to the right are good electron acceptor. These chemical descriptors have been used successfully in different chemical systems [59–66].

Binding energies (BE) were obtained as follows:

$$BE=\left[E\left(X\right)+E\left(Y\right)\right]-[E(XY)]$$

*X* represents PCBs and PBDEs or their quinone derivatives, and *Y* represents guanine, deoxyadenosine, or GSH. Basis set superposition error (BSSE) was obtained according to the Counterpoise method of Boys and Bernardi [67, 68].

Condensed Fukui functions are calculated by the molecular fragment response approach using finite differences between the atomic charges of the systems [66–68]. The equations to calculate the condensed Fukui functions are the following:

$$\begin{array}{cc}\mathrm{Nucleophilic \;attack}& {f}_{k}^{+}={Q}_{k}^{-1}-{Q}_{k}^{0}\\ \mathrm{Electrophilic \;attack}& {f}_{k}^{-}={Q}_{k}^{0}-{Q}_{k}^{+1}\\ \mathrm{Neutral \;o \;radical \;attack}& {f}_{k}^{0}={~}^{1}\!\left/ \!{~}_{2}\right. [{Q}_{k}^{+}+{Q}_{k}^{-}]\end{array}$$

*Q* are Mulliken charges of atom *k*. The larger the absolute value of *f*, the more reactive the atom will be.

## Results and discussion

**Fig. 3:**
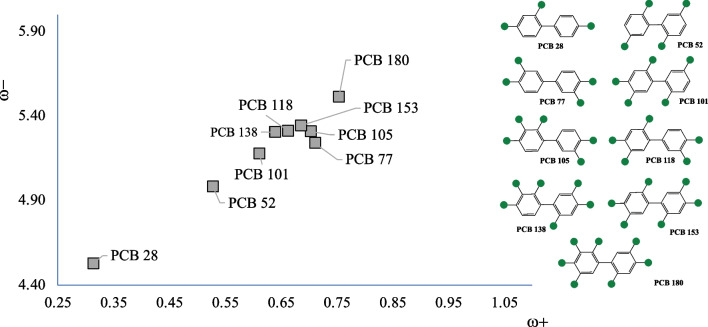
Electron donor–acceptor map (DAM) of PCBs. Values in eV

**Fig. 4:**
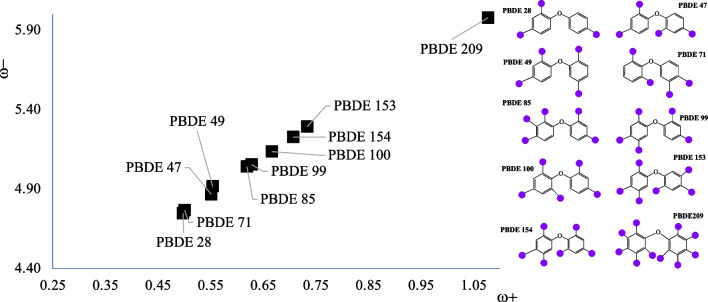
Electron donor–acceptor map (DAM) of PBDEs. Values in eV

To investigate the potential oxidant capacity of PCBs and PBDEs, the analysis of the electron donor–acceptor properties was realized using the DAM for each system. Figures 3 and 4 present the corresponding DAMs. The first thing to note is that the electron acceptor capacity increases with the number of Cl or Br atoms. The best electron acceptors are those located up to the right, and they are molecules with seven Cl atoms or ten Br atoms. The best electron donors are those with three Cl or Br atoms. This is consistent with the electronegativity of Cl and Br. They are more electronegative than H or C, and therefore, the presence of these halogens improves the electron accepting capacity. PCB-77, one of the most toxic compounds, is found in the middle of the DAM. It is not the best electron donor, nor the worst, and the same can be said for its electron acceptor capacity. Therefore, the greatest toxicity of this compound is not related to the ability to increase oxidative stress through the electron transfer mechanism. It was recently demonstrated [16] that PCB-28 stimulates oxidative stress and induced toxicity on plants. Results reported in Fig. 3 show that this compound is the best electron donor and therefore the best reductant molecule among all the PCBs under study. It donates an electron and is oxidized, and this could be related to oxidative stress. PCB-180 is the best electron acceptor and, therefore, the best oxidant.

Results of Fig. 4 indicate that PBDE-28 is the best electron donor. One of the most toxic compounds is PBDE-47, and it is in the middle of the DAM; it is not the best or the worse, electron donor or acceptor. Also in this case, there is no correlation between the electron transfer capacity and the potential toxicity. PBDE-209 is a potent oxidant that produces oxidative stress, and this is in agreement with the results of Fig. 4 since it is the best electron acceptor. Being a good electron acceptor implies that the molecule oxidized other compounds. PBDE-209 accepts electrons that another molecule loses.

**Fig. 5:**
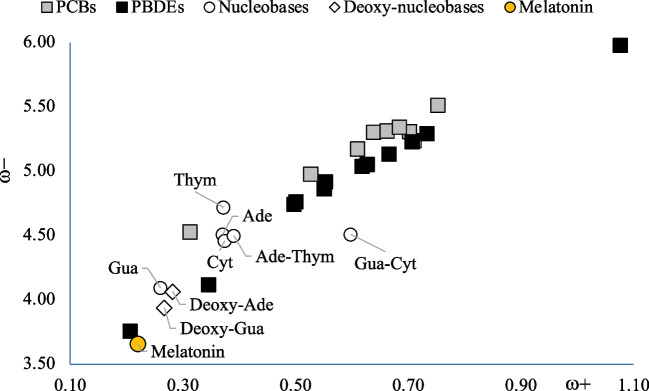
Electron donor–acceptor map (DAM) of PCBs (gray squares) and PBDE (black squares) including DNA nitrogen basis (white circles and rhombus) and melatonin (yellow circle). Values in eV

To analyze the possibility to donate or accept electrons, it is important to compare with the electron transfer capacity of DNA nitrogen basis. To this end, Fig. 5 presents the DAM including nitrogen bases. All biomolecules are located down to the left with respect to PCBs and PBDEs. Electrons will be transfer from molecules located down to the left (good donors) to those ubicated up to the right (good acceptors). Therefore, nitrogen basis will donate electrons to the persistent organic pollutants. This implies that these biomolecules will be oxidized by these compounds. PBDE-209 may be the most toxic since it is the best electron acceptor, and PCB-28, that is the worse electron acceptor, should be the less toxic.

Figure 5 includes results for melatonin. Melatonin is an antioxidant that was reported as potential antidote for PBDE-47 poisoning [16, 69]. Apparently, melatonin is able to protect cells from oxidative DNA damage induced by PBDE-47. Analyzing the capacity to donate or accept electrons of melatonin, the values in Fig. 5 indicate that it is the best electron donor and the worse electron acceptor of all molecules included. It is a good antioxidant molecule since it is the best electron donor. Melatonin prevents the oxidation of nitrogenous bases because it is oxidized first since it is a better electron donor. Oxidation is the loss of electrons; melatonin donates electrons and with that it is oxidized. These results explain the ability of melatonin to prevent oxidation and DNA damage.

**Fig. 6:**
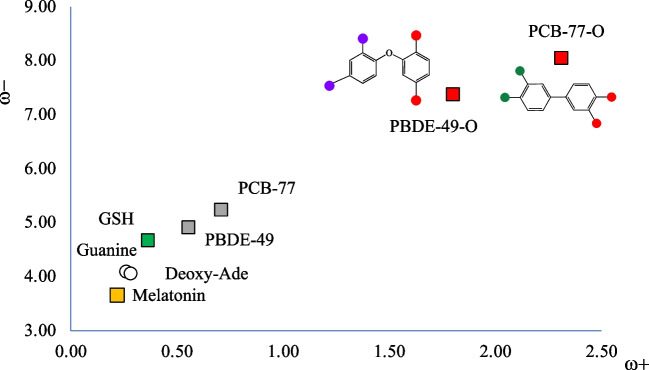
Electron donor–acceptor map (DAM) of quinones (red squares, PCB-77-O and PBDE-449-O). Correspondent PCB and PBDE (gray squares); guanine, deoxy-adenine (white circles), and melatonin (yellow square) are included for comparison. Values in eV. Red dots in molecular structures represent oxygen atoms

To investigate the potential toxicity of quinone-type derivatives, two examples were selected: PBC-77-O and PBDE-79-O. The DAM of these compounds is presented in Fig. 6. A schematic representation of these molecules is also included. It was reported that quinone-type derivatives are more toxic for two reasons: they have redox properties that increases oxidative stress and can react with GSH and DNA [39]. Redox properties can be analyzed with the DAM reported in Fig. 6.

It is clear that quinone-type derivatives are the best electron acceptor molecules, and this explains the redox toxicity. They increase the oxidative stress accepting electrons from other molecules. Melatonin in this case also protects against the oxidative stress that quinone-type derivatives may produce. The results of Fig. 6 explain the greater toxicity of these compounds.

Throughout all the research reported until now, it has become completely clear that DNA oxidation is an important consequence of life in an oxygen-rich atmosphere. Survival in the presence of oxygen is possible thanks to DNA repair enzymes. However, when further oxidation occurs due to the presence of contaminants such as these persistent organic molecules, the enzymes are not able to repair the DNA, and mutations can occur. PCB-28, PCB-118, PCB-153, PBDE-153, and PBDE-209 are the most abundant compounds found in Antarctic penguins [3]. It was previously reported [70] the genetic damage in different species of Antarctic penguins by microscopic observation of erythrocytic nuclear abnormalities. The information reported provided the reference data. It will be for future work to investigate the genomic damage related to the presence of PCBs and PBDEs.

Substances that are genotoxic may bind directly to DNA or act indirectly leading to DNA damage by affecting enzymes involved in DNA replication, thereby causing mutations which may or may not lead to cancer or birth defects (inheritable damage). To investigate the direct interaction of these persistent organic pollutants, three examples of each family were selected: the best electron donor, the best electron acceptor, and the most toxic according with the reported data. Guanine was used as an example of nitrogen bases. Deoxyadenosine was also used since it has been reported to be a good model for studying the interaction of toxic molecules with DNA and determining potential genotoxic effects [66]. Results are reported in Table 1. For PCB-77, there are previous theoretical results [44] that are in agreement with those reported in this investigation.

**Table 1:**
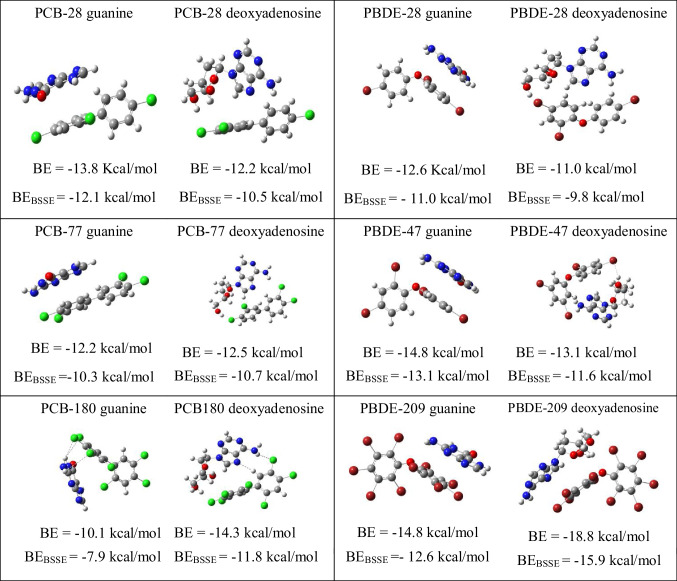
Optimized structures and binding energies of the interaction between DNA nitrogen bases and different PCBs and PBDEs. BE_BSSE_ is the binding energy including basis set superposition error (BSSE)

According to the binding energies, all interactions are energetically favorable; products are more stable than reactants. The binding energies of the three PCBs are similar. PCB-77 was reported as the most toxic, but the results of this investigation indicate that the binding energy is similar to the others PCBs. This suggests that the high toxicity is not due to the direct interaction with DNA basis. For PBDEs, the best electron acceptor also presents the highest binding energy. These results do not explain why PBDE-28 is more toxic than PDBE-209. Apparently, better electron acceptors will interact stronger than a good electron donor.

In any case, hydrogen bonds are formed between biomolecules and PCBs or PBDEs, and the binding energies of all systems indicate that these molecules can interact directly with the nitrogenous bases of DNA. This suggests a possible explanation for the highly carcinogenic properties of these chemical compounds. More studies are necessary to analyze other reaction mechanisms that can provide more explanations about the toxicity, but as a consequence of this research, it can be said that these persistent organic pollutants are dangerous due to the oxidative stress they can produce and because they can interact directly with the nitrogenous bases of DNA. Furthermore, it is possible to conclude that those persistent organic pollutants that have more halogen atoms that replace hydrogen are potentially more toxic.

**Scheme 1.**
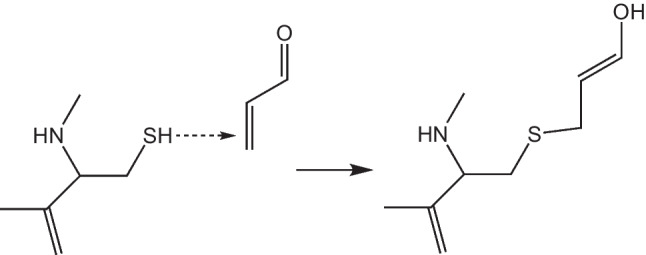
Representation of the Michael-type addition

To investigate the reaction of quinone-type derivatives, we also investigate its interaction with guanine and deoxyadenosine. The reaction with GSH is also analyzed because it was reported that quinones interact with GSH with a Michael-type addition. Michael-type addition is represented in Scheme 1. Results are reported in Table 2.

**Table 2:**
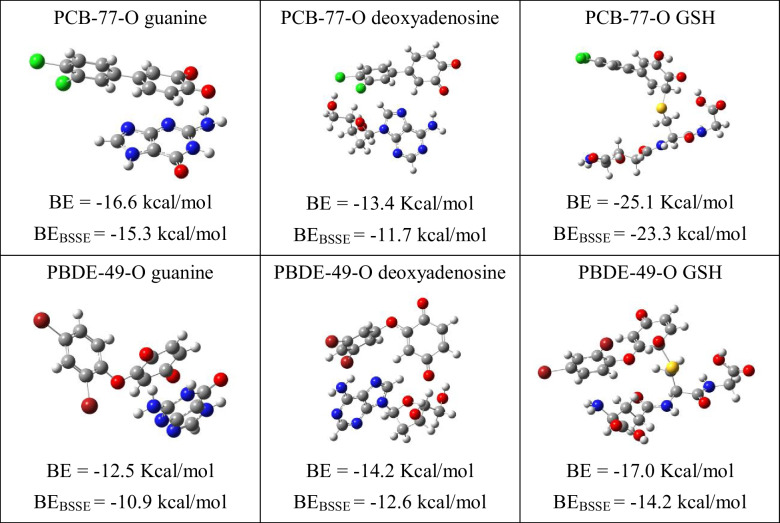
Optimized structures and binding energies of the interaction between GSH and DNA nitrogen bases with two quinones (PCB-77-O and PBDE-49-O)

The binding energies of quinone-type derivatives indicate that the interaction is energetically possible, the strongest being with GSH. With GSH, the binding energy is larger for PCB-77-O than for PCB-49-O, and therefore, the first is potentially more dangerous than the second. PCB-77-O is also the best electron acceptor. This corroborates our hypothesis that the best electron acceptors will interact stronger than the best electron donors. These results suggest that quinone-type derivatives are potentially more toxic than PCBs and PBDEs, and this is in complete agreement with previous results reported until now.

**Fig. 7:**
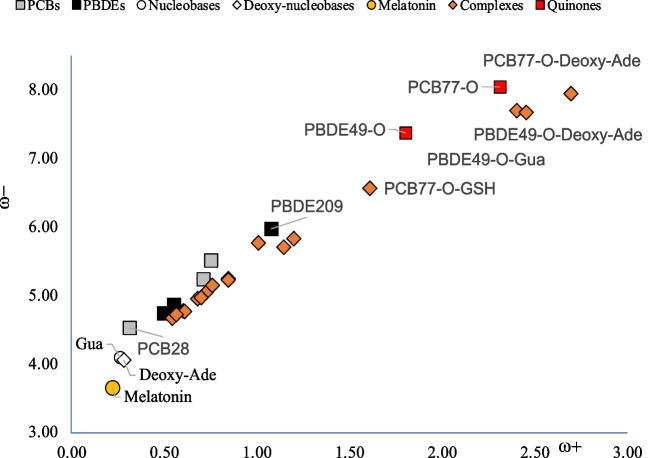
Electron donor–acceptor map (DAM) of quinones (red squares, PCB-77-O and PBDE-49-O). Correspondent PCB and PBDE (gray and black squares). Complexes are in orange rhombus. Guanine, deoxy-adenine (white circles), and melatonin (yellow square) are included for comparison. Values in eV

To analyze the electron transfer capacity of the complexes, Fig. 7 reports the corresponding DAM. All complexes have similar values to PCBs and PBDEs, with the best electron acceptors being those formed with quinone-type derivatives.

The electro-donating and electro-accepting powers are global chemical reactivity descriptors. To have more information about the reactivity of these persistent organic pollutants, it is important to analyze the reactivity at the local level. Local reactivity parameters are useful to differentiate the reactivity of atoms that form molecules. The Fukui function is one of the most used local reactivity parameters. It is associated with the response of the electron density to a change in the number of electrons, with the external potential remaining constant. Condensed Fukui functions are calculated according to the equations presented previously.

Two systems were analyzed: PCB-77 and PBDE-49. The corresponding quinone derivatives were also investigated. The results are presented in Table 3. The condensed Fukui functions indicate that the electrophilic attack will occur on choro or bromine atoms, while the neutral or radical attack will occur on oxygen atoms of the quinone-type derivatives. This is important and may be useful in explaining previous experimental results indicating greater toxicity of quinone-type derivatives than of PCBs or PBDEs. Quinone-type derivatives can react with free radicals. The consequences of this will have to be investigated in the future.

**Table 3:**
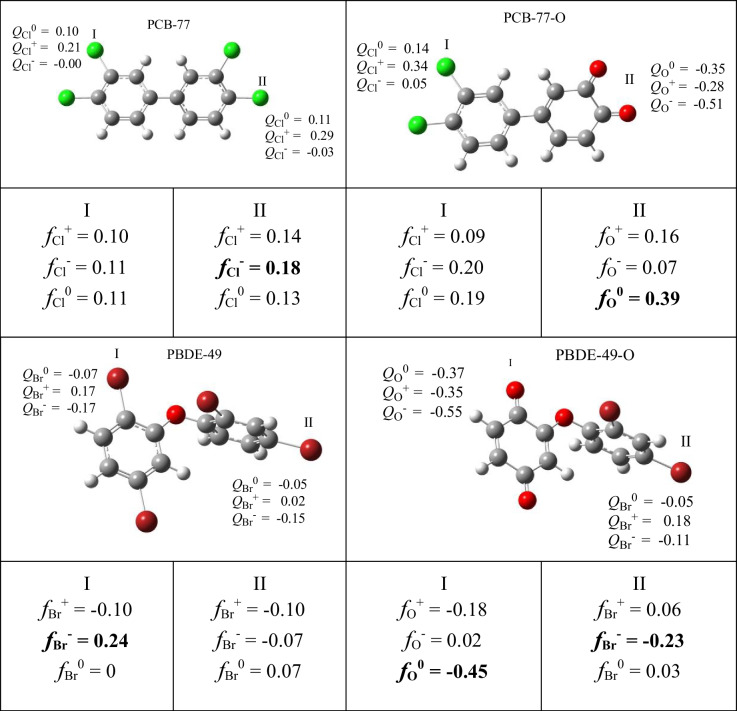
Condensed Fukui functions of PCB-77, PCB-77-O, PBDE-49, and PBDE-49-O

## Conclusions

The electron transfer properties of PCBs and PBDEs show that the number of halogens is directly related to the ability to accept electrons. The more chlorine or bromine atoms there are, the more capacity the molecules will have to accept electrons. Molecules that accept electrons are oxidants, since they accept electrons that other molecules lose. Therefore, molecules with more hydrogens substituted by halogens are expected to be more toxic due to the greater oxidative stress they can produce. Quinone-type derivatives are the best electron acceptors among all the compounds studied, and therefore, they are potentially the most toxic. Quinone-type derivatives increase oxidative stress by accepting electrons.

Melatonin is an antioxidant that was reported as potential antidote for PBDE-47 poisoning. The results reported here reveal that melatonin prevents the oxidation of nitrogen bases that PCBs, PBDEs, and quinone-type derivatives may produce, by donating electrons. It is oxidized first since it is a better electron donor. Oxidation is the loss of electrons; melatonin donates electrons and with that it is oxidized. These results explain the ability of melatonin to prevent oxidation and DNA damage.

PCBs, PBDEs, and quinone-type derivatives are toxic because of their electron donor-accepting properties that are related to oxidative stress and because they could bind directly to the nitrogenous bases of DNA. Quinone-type derivatives interact stronger than PCBs and PBDEs with DNA nitrogen bases, and they also interact strong with GSH, in agreement with the experiments. This increases the potential toxicity of quinone-type derivatives. The condensed Fukui functions indicate that the electrophilic attack will occur on choro or bromine atoms, while the neutral or radical attack will occur on oxygen atoms of the quinone-type derivatives. The importance of this research is that it provides information that can be used together with other experiments and calculations, to analyze possible mechanisms that explain the toxicity of these compounds.

## Data Availability

No datasets were generated or analyzed during the current study.
